# Improving heterologous membrane protein production in *Escherichia coli* by combining transcriptional tuning and codon usage algorithms

**DOI:** 10.1371/journal.pone.0184355

**Published:** 2017-09-13

**Authors:** Nico J. Claassens, Melvin F. Siliakus, Sebastiaan K. Spaans, Sjoerd C. A. Creutzburg, Bart Nijsse, Peter J. Schaap, Tessa E. F. Quax, John van der Oost

**Affiliations:** 1 Laboratory of Microbiology, Wageningen University and Research, Wageningen, The Netherlands; 2 Laboratory of Systems and Synthetic Biology, Wageningen University and Research, Wageningen, The Netherlands; 3 Institut für Biologie II, Albert Ludwigs Universität Freiburg, Freiburg, Germany; Tel Aviv University, ISRAEL

## Abstract

High-level, recombinant production of membrane-integrated proteins in *Escherichia coli* is extremely relevant for many purposes, but has also been proven challenging. Here we study a combination of transcriptional fine-tuning in *E*. *coli* LEMO21(DE3) with different codon usage algorithms for heterologous production of membrane proteins. The overexpression of 6 different membrane proteins is compared for the wild-type gene codon usage variant, a commercially codon-optimized variant, and a codon-harmonized variant. We show that transcriptional fine-tuning plays a major role in improving the production of all tested proteins. Moreover, different codon usage variants significantly improved production of some of the tested proteins. However, not a single algorithm performed consistently best for the membrane-integrated production of the 6 tested proteins. In conclusion, for improving heterologous membrane protein production in *E*. *coli*, the major effect is accomplished by transcriptional tuning. In addition, further improvements may be realized by attempting different codon usage variants, such as codon harmonized variants, which can now be easily generated through our online Codon Harmonizer tool.

## Introduction

Throughout the three domains of life (eukarya, bacteria and archaea), 15–30% of all genes encode integral α-helical membrane proteins [1]. This diverse group of proteins is involved in a variety of crucial processes, such as energy transduction, transport and signaling. To characterize membrane proteins, for example by biochemical assays or protein structure crystallography, overproduction of membrane proteins in recombinant hosts, such as *Escherichia coli*, is a key method. Also for metabolic engineering and synthetic biology endeavors in *E*. *coli* and other relevant organisms, functional, recombinant expression of membrane proteins, including transporters, sensors and enzymes is of utmost importance. Additionally, 70% of all drugs target human membrane proteins, and heterologous expression of these proteins is a crucial step in drug discovery and development [2].

The recombinant production of membrane proteins, however, is often challenging, due to the fact that only low amounts of protein are properly folded and translocated into the membrane. Overproduced membrane proteins often end up as insoluble aggregates in the cytoplasm, accumulated in so-called inclusion bodies [3]. For *E*. *coli* it has been demonstrated that this phenomenon can be partly related to the jamming of the membrane translocation systems, such as the Sec-translocon [3]. To address these issues, some tools have been developed to improve membrane protein production, mostly for the common expression host *E*. *coli*. Several *E*. *coli* strains have successfully been optimized for membrane protein production, including the ‘Walker strains’, *E*. *coli* C41(DE3) and C43(DE3) [4], and *E*. *coli* LEMO21(DE3) [5]. These strains are all based on reducing the high transcription rates from the T7 RNA polymerase (T7RNAP), which is commonly used in *E*. *coli* (DE3) strains to drive recombinant gene expression. The improved membrane protein production levels in these Walker and LEMO strains, rely respectively on reduced expression of T7RNAP [6] or on fine-tuning of the expression level of T7RNAP (S1 Fig) [5]. The protein production improvements of these strains are related to tuning the transcription rates of the recombinant mRNA, which can help to prevent the overload of chaperones and membrane insertion machineries.

On a translational level, codon usage also plays a key role for functional recombinant protein production. The fact that different organisms use different synonymous codons, is important to take into account when overexpressing heterologous proteins [7]. To overcome problems in the expression of mostly eukaryotic genes, other *E*. *coli* strains have been developed, such as the Rosetta strains, which overexpress tRNA species for codons that are rare in *E*. *coli* [8].

In recent years, synthesized gene sequences with adapted codon usages have become another important tool to attempt to improve recombinant expression [9]. Hereto, typically coding regions are optimized, mostly by proprietary algorithms of commercial vendors, through mainly selecting codons that occur frequently in the expression host. It has to be noted that different optimization algorithms apply different methods to determine the codon frequencies in the expression host, for example based on codon usage in all protein-coding genes or only for a limited set of highly expressed genes; as another alternative, preferred codons are determined based on their cognate tRNA gene copy numbers in the expression host [10]. In addition, most of the codon optimization algorithms are multi-parameter algorithms, taking into account several other factors as well. These include aiming for a desired GC-content, avoiding strong mRNA secondary structures in the 5’UTR, and avoiding of certain undesired motifs, such as repeats, Shine-Dalgarno like sequences and RNase sites [9,11]. Even though there is a large variety in available codon optimization algorithms, the recurring motif is their preferred use of frequent host codons. In this study the commonly applied, proprietary GeneOptimizer Algorithm from GeneArt is employed, which is a multi-parameter algorithm taking into account frequent host codon usage, GC content and several other parameters [12,13].

Recent experimental and bioinformatics analyses of codon usage within genes have revealed that ‘rare’ codons can have an important role in functional production of proteins [7,14–18]. Rare codons are hypothesized to slow down translation in order to accommodate proper folding of certain protein domains, such as α-helices and β-sheets [14]. Also for membrane proteins, it has been suggested that clusters of rare codons may provide translational pauses that facilitate co-translational folding of specific domains and membrane insertion [19]. Rare clusters of codons in genes encoding membrane proteins, e.g. in *Saccharomyces cerevisiae*, have been correlated to the translocation of membrane proteins [20]. The best algorithm so far, which takes the importance of rare codons into account, is the so-called ‘codon harmonization’ algorithm [21,22]. This algorithm ensures that the frequency of a codon in the expression host, selected for the synthetic coding sequence, is similar to the frequency of the original codon in the wild-type gene sequence in the native host. A few variants of harmonization algorithms have been proposed, including algorithms with minimum thresholds for very rare codons [22], or a harmonization algorithm based on the tRNA gene copy numbers of native and expression hosts as an alternative to native and expression host codon usage frequencies [15]. However, the main principle of all harmonization algorithms is to mimic native codon usage, including more rare codons, which is a fundamentally different principle than applied in codon optimization algorithms.

Algorithms based on harmonization have been applied for heterologous expression of a few eukaryotic and bacterial cytoplasmic and membrane proteins in *E*. *coli* and *S*. *cerevisiae*. In several cases it was reported that the codon harmonized variant gave increased heterologous production compared to production from the wild-type sequence variants [18,21,23–25]. Apart from causing higher production levels, some studies that compare harmonized with wild-type or optimized gene variants report higher specific activities after expressing proteins from harmonized genes, presumably due to better folding [15,18,26].

So far, the general performance of this codon harmonization algorithm on membrane protein production in *E*. *coli* has not been studied elaborately; no studies have compared the production levels of several membrane proteins from different native organisms. Furthermore, apart from the single-gene study of Vuoristo *et al*. [26], no other studies compared the performance of the codon harmonization algorithm with a typical codon optimization algorithms. Therefore, in the current study the membrane-integrated production of 6 membrane proteins is analyzed, including some difficult-to-express membrane proteins. To this end, codon-harmonized, codon-optimized, and wild-type coding variants of the genes were fused to Green Fluorescent Protein (GFP) at their C-termini for easy-monitoring of membrane-integrated production in *E*. *coli* [27]. In addition, expression of all these variants was fine-tuned on a transcriptional level using the *E*. *coli* LEMO21(DE3) strain. To ensure a more widespread, convenient availability of the here employed codon harmonization algorithm throughout the scientific community, we developed the online accessible, user-friendly Codon Harmonizer tool.

## Results and discussion

### Applying harmonization and optimization algorithms

Six different integral membrane proteins were selected from bacteria, archaea and eukarya, to compare their heterologous, membrane-embedded production in *E*. *coli* from wild-type, optimized and harmonized gene variants (Table 1). Four of the selected membrane proteins are light-harvesting proton-pumping rhodopsins (PPRs) originating from all domains of life. PPRs are membrane proteins that harbor 7 transmembrane domains and covalently bind a retinal pigment. The retinal pigment here functions to absorb a photon, leading to a conformational change of the pigment, which eventually leads to a proton being extruded from the cell, resulting in a proton motive force. These PPRs were specifically targeted in this study as they can function as simple energy-harvesting photosystems in many organisms, and through heterologous expression they can serve wide applications, which include optogenetic sensors in neuroscience [28] and optogenetic control or light-driven ATP regeneration in microorganisms [29]. Some of the selected PPRs have already been expressed relatively successfully in *E*. *coli*, such as bacterial *Gloeobacter violaceus* rhodopsin (GR) [30,31] and archaeal *Haloarcula marismortui* rhodopsin (HR) [32], while others are not (yet) expressed in *E*. *coli* to appreciable levels, such as bacteriorhodopsin (BR) [33] and leptosphaeria rhodopsin (LR) [34].

**Table 1:** Overview of all 6 tested membrane proteins for which the expression of different gene variants was studied and their analyzed codon usage parameters.

*Gene*	*Protein*	*Native host*	*Domain*	*# TM-domains*	*CHI*^1^ *for E*. *coli*			*CAI*^2^ *for E*. *coli*			*CAI native host WT*	*Refs*
					*HA*	*OP*	*WT*	*HA*	*OP*	*WT*		
GR	gloeobacter rhodopsin	*Gloeobacter violaceus*	Bacteria	7	0.099	0.339	0.221	0.583	0.869	0.606	0.499	[30]
BR	bacteriorhodopsin	*Halobacterium salinarum*	Archaea	7	0.057	0.213	0.234	0.762	0.897	0.687	0.678	[33]
HR	halorhodopsin	*Haloarcula marismortui*	Archaea	7	0.041	0.190	0.219	0.761	0.901	0.688	0.738	[32]
LR	leptosphaeria rhodopsin	*Leptosphaeria maculans*	Eukarya	7	0.066	0.183	0.279	0.813	0.869	0.607	0.823	[34]^3^
NorB	nitric oxide reductase	*Moraxella catarrhalis*	Bacteria	14	0.083	0.197	0.250	0.787	0.888	0.658	0.723	[35,36]
DGGGPs	2,3-di-O-geranyl-geranyl-glycerylphosphate synthase	*Methanococcus maripaludis* C5	Archaea	7	0.056	0.230	0.281	0.743	0.913	0.542	0.698	[37]

Abbreviations: CHI: Codon Harmonization Index; CAI: Codon Adaptation Index; WT: wild-type; HA: harmonized; OP: optimized; TM-domains: Trans-Membrane domains

Color shading; dark green: variant that produces significantly higher than the lowest producing variant(s) for that protein; light green: variant that produces both significantly higher than the lowest producing variant and significantly lower than the highest producing for that protein; grey: variant that produces significantly lower than the highest producing variant(s), significances based on two-tailed, unpaired t-tests with unequal variances, p<0.05, non-shaded CAI and CHI cells indicate no significant differences among the production levels of the variants for those proteins

^1^ $CHI=\frac{1}{N}\sum_{i=1}^{N}\text{abs}(RCA_{i}-RCA_{i,native}),$ in which RCA_i_ denotes the relative codon adaptiveness of the i^th^ codon, RCA_i,native,_ the relative adaptiveness of the i^th^ native codon in the native host, and N the number of codons in the gene

^2 $CAI={\left(\Pi_{i=1}^{N}\;RCA_{i}\right)}^{\frac{1}{N}},$^ in which RCA denotes the relative codon adaptiveness of the i^th^ codon in a gene and N the number of codons in the gene

^3^not expressed before in *E*. *coli*

In addition to PPRs, we tested two different integral membrane enzymes from different domains of life. Nitric oxide reductase (NorB) from the bacterium *Moraxella catarrhalis*, for which it was shown previously that the GeneArt codon-optimized variant in *E*. *coli* resulted in a significantly reduced production level compared to production of the wild-type gene in *E*. *coli* [35]. Furthermore, we included an archaeal 2,3-di-*O*-geranyl-geranyl-glycerylphosphate synthase (DGGGPs) from *Methanococcus maripaludis*, an enzyme catalyzing ether bond formation in archaeal lipid biosynthesis. Successful heterologous production of this integral membrane enzyme has been a major challenge for the transfer of the archaeal lipid biosynthesis pathway to the bacterium *E*. *coli* [38].

**Fig 1:**
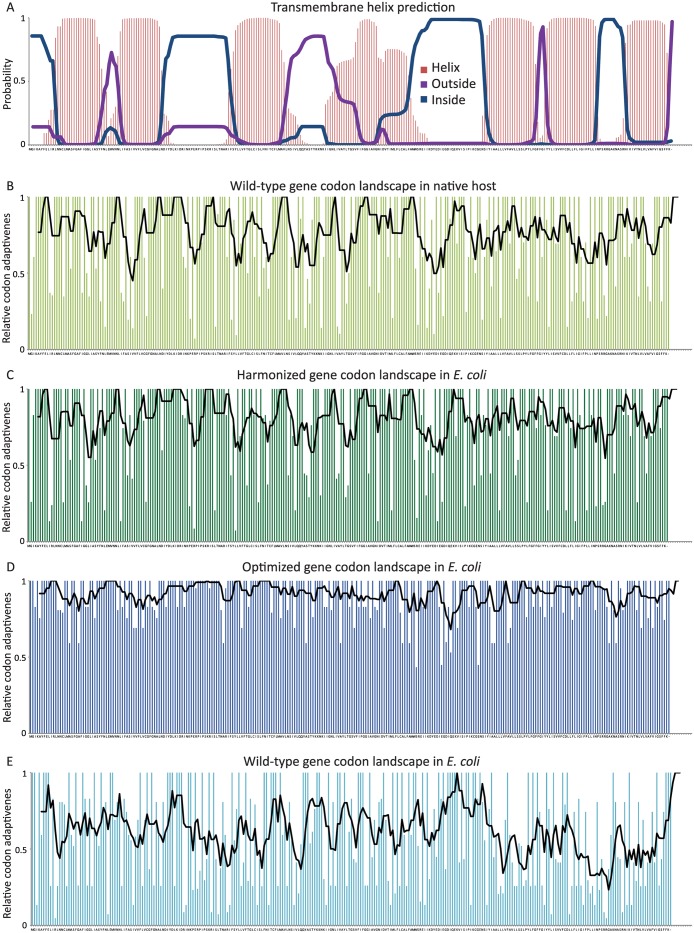
Transmembrane helix prediction and codon usage landscapes for the different variants for DGGGPs. (a) Transmembrane helix prediction plot depicting the probability of residues being in a transmembrane helix domain (red bars), on the inside or cytosolic side of the membrane (blue line) or outside of the membrane (purple line) ((TMHMM v2.0). Codon usage landscapes are depicted based on Relative Codon Adaptiveness (RCA) scores for individual amino-acids and a moving average over 5 codons (black line), for (b) the wild-type gene for native host codon usage (*M*. *maripaludis* C5); (c) the codon-harmonized gene variant for *E*. *coli* codon usage; (d) the codon-optimized gene variant for *E*. *coli* codon usage (e) the wild-type gene variant for *E*. *coli* codon usage.

For all 6 gene candidates, a codon harmonized sequence was generated by our online Codon Harmonizer tool, and a codon-optimized sequence was obtained from the proprietary GeneOptimizer Algorithm from GeneArt. The codon harmonization we performed was based on the harmonization algorithm originally proposed [21,22]. Our Codon Harmonizer tool generates harmonized sequences, using the codon usage frequency tables for the native and expression host, based on all codons in the protein-coding genes annotated in NBCI genome assemblies as inputs. The algorithm then selects the codons for the synthetic sequence to most closely match the native codon frequency usage. For all the tested genes so-called ‘codon frequency landscapes’ were generated. As intended, the codon landscapes of the harmonized variants for *E*. *coli* are comparable to the landscapes of the wild-type variants for the native host (Fig 1 and S2 Fig). Apart from assessing the codon landscapes graphically, they can also be evaluated quantitatively based upon a proposed Codon Harmonization Index (CHI). A CHI value close to 0 indicates a well-harmonized gene, all harmonized variants in this study have a CHI <0.1 (Table 1). All codon-optimized and wild-type variants have codon landscapes in *E*. *coli* that deviate further from the native codon landscape and consequently their CHI has higher values than those for the harmonized variants (≥0.183). Especially the wild-type variants of archaeal DGGGPs and eukaryotic LR have high CHI scores (≥0.279).

The landscapes of the codon-optimized variants for *E*. *coli* generally form a ‘high plateau’, because they mainly contain frequent codons (Fig 1 and S2 Fig). However, due to some additional rules of the GeneOptimizer multi-parameter algorithm, or potentially due to a different, proprietary codon frequency table for *E*. *coli*, also some codons with an apparent lower frequency are occasionally included. Nevertheless, rare codons are hardly appointed by thisa algorithm, in sharp contrast to the harmonization algorithm. The preference for frequent codons in codon-optimized variants is also reflected by the high Codon Adaptation Index (CAI) scores, calculated from codon usage for all genes, which are all above 0.869. The unmodified wild-type gene variants from the original organisms, have expectedly lower CAI scores based on *E*. *coli* codon usage; especially the wild-type sequences of the eukaryotic and archaeal genes result in lower CAI values (≤0.542) (Table 1). The harmonization algorithm mostly increases CAI scores compared to the wild-type variants, but generally to a lower extent than the optimization algorithm. Lower CAI increases from the harmonization algorithm are expected, as this algorithm deliberately includes rare codons, reducing CAI, to mimic the codon landscape of the wild-type gene in the native host.

### Transcriptional tuning significantly improves membrane protein production levels

To allow for easy quantification of membrane-integrated protein levels, GFP was used as a reporter for membrane-embedded proteins and monitored by whole-cell fluorescence assays [27,39]. The GFP protein was fused to the C-terminus of the membrane proteins. GFP will generally only be folded properly and generate a fluorescent signal when the fused membrane protein is integrated into the membrane [39]. This method only works for membrane proteins with intracellular C-termini. However, an alternative method is available for membrane proteins with an extracellular C-terminus, in this work employed for DGGGPs. Hereto the pWarf vector was applied, which fuses an additional single transmembrane spanning domain in between the extracellular C-terminus of DGGGPs and the GFP-fusion [40]. This allowed for intracellular localization and proper folding and fluorescence of the GFP reporter domain.

For all membrane proteins and their three codon variants, it was tested by in-gel fluorescence if the GFP signal originated from a single fusion protein (S3 Fig). Except for LR, for all the proteins the fluorescence signal originates primarily from a single fusion product of the correct size. Hence for all proteins tested, with the exception of LR, we could use whole-cell fluorescence to properly assess membrane-integrated production.

**Fig 2:**
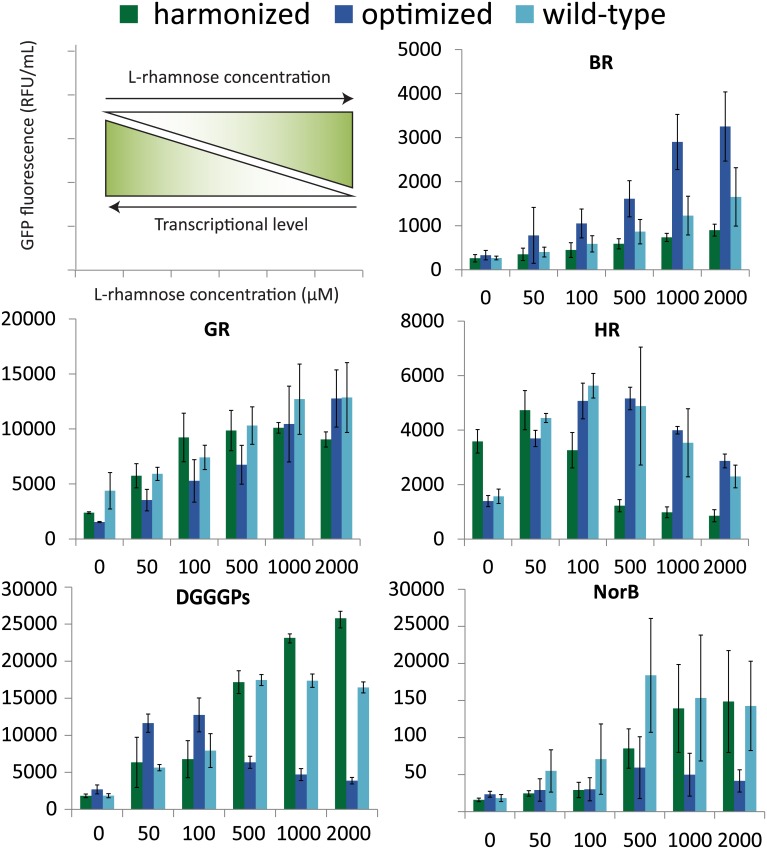
Membrane-integrated production levels for all codon usage variants. Production levels in *E*. *coli* LEMO(DE3) were determined by whole-cell GFP-fluorescence at different transcriptional tuning by varying the L-rhamnose concentration (indicated in μM). All expression experiments were at least performed in biological triplicates.

Expression by the *E*. *coli* LEMO(DE3) strain allowed us to optimize the level of the integrated-membrane proteins of interest by transcriptional tuning by varying L-rhamnose in the common range (for mechanism see S1 Fig). We observed for all proteins and variants that adding a certain amount of L-rhamnose (i.e. moderate down-tuning of transcription) always resulted in significantly higher levels of fluorescence compared with adding no L-rhamnose (i.e. maximum transcription) (Fig 2). For the wild-type codon variants of all membrane proteins, optimization by transcriptional tuning led to 2–10 times improved production. Also for the harmonized and optimized variants of those proteins, transcriptional tuning generally improved heterologous production by similar orders of magnitude. However, it has to be noted that the optimal level of tuning, i.e. optimal concentration of L-rhamnose, frequently differs among different codon usage variants for the same protein. Previously, it was already demonstrated that the optimal level of transcription is specific for different proteins and expression conditions [35], and as we demonstrate here, this is also true for different codon usage variants.

For most of the membrane proteins and their codon variants, (down-)tuning of transcription appeared important to reach the highest level of properly folded, membrane-integrated protein. The role of tuning presumably lies in properly matching translation rates with the folding and translocation rates of heterologous protein into the *E*. *coli* membrane. This may facilitate proper integration into the membrane and avoid accumulation in inclusion bodies. One could expect that GeneArt codon-optimized gene variants, which consist mainly of frequently used codons, have the fastest translation rates and hence require more down-tuning. Consequently one could expect that the codon-optimized variants generally require higher L-rhamnose concentrations for optimal production; however this relation cannot be clearly observed for most genes. This may be due to the fact that also other factors determine the translation rates of different variants, such as the translation initiation rates and mRNA stability of different variants.

Interestingly, for membrane proteins that have been reported to be hard-to-express in *E*. *coli* such as DGGGPs and BR, it seems that tuning down to the lowest tested transcriptional level (2000 μM L-rhamnose) can substantially improve production levels. Down-tuning of transcription increased membrane-integrated production of the wild-type BR and wild-type DGGGPs by 6-fold and 10-fold, respectively. However, to (further) increase the production of these proteins and some others, it appears that the different codon usage variants can play a role as well.

### Different codon usage algorithms improve production of some membrane proteins

**Fig 3:**
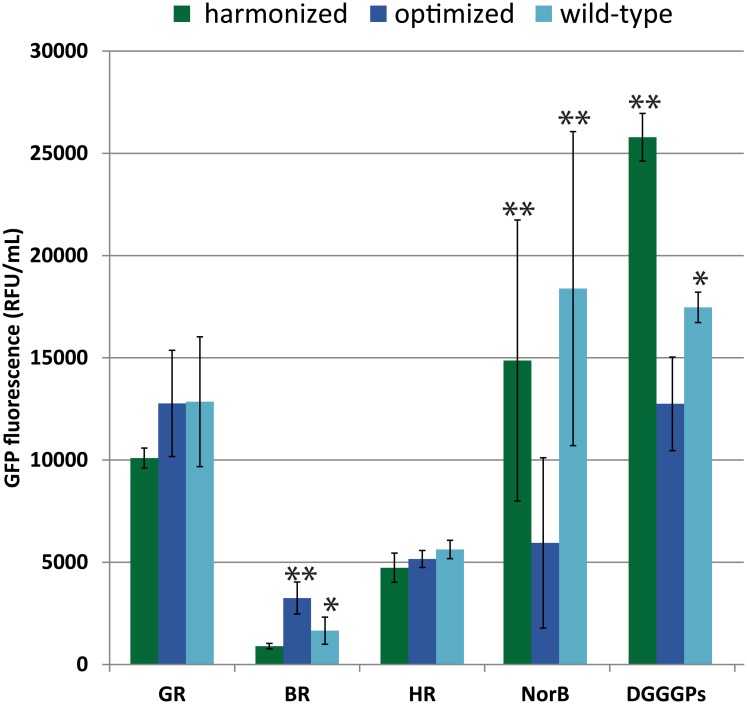
Comparison of the highest membrane-integrated production levels for different codon usage variants. All expression experiments were at least performed in independent triplicates. * indicates this variant is produced both significantly higher than the lowest producing variant and significantly lower than the highest producing variant for that protein (two-tailed, unpaired t-tests with unequal variances, p<0.05). ** indicates the production levels of this or these variants are significantly higher from the lowest producing variant(s) for that protein (two-tailed, unpaired t-tests with unequal variances, p<0.05).

In this study we compared the influence of three different codon usage variants on production levels of membrane-integrated proteins. After optimization by transcriptional tuning, maximum achieved production levels for each codon usage variant were compared. This gave rather mixed results on the success of different codon usage variants for different membrane proteins (Fig 3). For most PPRs there was no large or no significant difference in the maximum production level between wild-type, harmonized or optimized variants. For the fungal PPR LR, the whole-cell fluorescence data are not reliable because the signal seems to be dominated by unfused “loose” GFP instead. However, in-gel fluorescence of the specific LR-GFP band indicates low, but clearly visible levels of LR-GFP fusion production for both the harmonized and optimized variant, while this band is hardly detectable for the wild-type variant (S3 Fig). This indicates that for low-level production of this eukaryotic PPR, both the optimized and harmonized variant are beneficial when compared to the wild-type variant. Furthermore it seems that the optimized variant expresses better than the harmonized variant, but from the in-gel fluorescence for LR it is hard to quantitatively determine if those two adapted codon variants give significantly different results.

For BR a further improvement is achieved, on top of the tuning, by applying the codon-optimized variant, resulting in a further doubling of the production level compared to using a wild-type variant. Surprisingly, for BR, the harmonized variant did not improve the production compared to the wild-type variant, but instead decreased the production level slightly. So for hard-to-express BR, combining codon-optimization and transcriptional tuning seems to be the best strategy to improve the production, however compared to other proteins its production level is still very low.

For the other two, non PPR, membrane proteins it was shown that the harmonization algorithm can be a fruitful strategy to increase membrane-integrated protein production. For both DGGGPs and NorB, the GeneArt codon-optimized variants resulted in significantly reduced production compared to the wild-type variants. As was already observed for some other membrane proteins [13,35], codon optimization may result in reduced membrane-integrated production, possibly due to less efficient folding and/or translocation resulting from the dominant usage of frequent codons. This decrease in production for the codon optimized variants for these two proteins could be counteracted by the codon harmonized variants. Harmonization restored the heterologous production of NorB to a similar level as the wild-type variant. More interestingly, codon harmonization of the DGGGPs gene improved the production further by almost 50% compared to the wild-type variant.

As a general observation, we note that harmonization is beneficial for increasing membrane-embedded production compared to wild-type variants for some proteins, such as LR and DGGGPs, for which in this study the wild-type CHI score is also highest (≥0.279). This suggest that especially for cases in which the codon landscape of the wild-type gene in *E*. *coli* deviates largely from the landscape in the native hosts, harmonization seems to be a promising approach for improved membrane protein production.

## Conclusion

Here we demonstrate, by using a set of different membrane proteins, that a combination of transcriptional tuning and different codon usage variants can be a successful approach to improve heterologous membrane protein production. Transcriptional tuning was demonstrated to be the most important factor for improving production of all tested membrane proteins, while applying different codon usage variants gave mixed results.

The used GFP-folding reporter approach has been instrumental for this work and allowed for a convenient quantitative screen of membrane-integrated production for most of the proteins at different tuning-conditions. However, this approach is not suitable for accurate determination of production levels for all proteins, such as observed for LR-GFP. Though this study was limited to observing protein production in LB medium at 37°C, other conditions, such as commonly applied lower temperatures (20–30°C) or other induction protocols, could be assessed further to determine optimal production conditions using the GFP-based screening as well. GFP-fusions generally seem to be a good proxy for membrane-integrated production; however, functional protein production levels for different codon variants and tuning conditions may be further studied by specific, quantitative protein activity assays if such assays are available.

For the codon usage algorithms, we expected that the relatively novel strategy of codon harmonization was a specifically promising strategy to improve the membrane-integrated production. The underlying rationale was that stretches of more rare codons in the gene in the native host play an important role in proper folding and subsequent translocation of membrane proteins. These processes are often regarded as most crucial for the successful production of membrane-integrated proteins. However, the mixed results of the codon optimization and harmonization algorithms for different proteins, again emphasize the complexity of optimizing codon usage for high-level protein production [41] and specifically for membrane protein production [19]. Codon usage and more general the mRNA sequence, have been shown to influence expression in many ways and new insights are still being elucidated [7,42]. In general, studies to determine the influence of codon usage on both native and heterologous gene expression show a great complexity and interrelatedness of involved factors, which may differ between different hosts, proteins and conditions. Important factors include frequent and rare codon usage, but also mRNA secondary structures, mRNA stability, concentrations of (charged) tRNA species, Shine-Dalgarno like sequences, co-occurrence of specific codons and many more factors [7]. Both the multi-parameter codon-optimization algorithm and, especially the harmonization algorithm, are based on simplified assumptions taking only a few of these factors into account. Harmonization as employed here, only takes into account whether specific codons in the wild-type gene are rare or frequent, relative to the overall codon usage in the native host. This codon frequency usage landscape is mimicked as close as possible by the harmonization algorithm, using the overall codon usage of the expression host *E*. *coli*. The harmonization approach could potentially be further improved by also optimizing for some other potentially important parameters, such as avoiding strong mRNA structures in the 5’UTR, as was shown to be useful for membrane proteins [43,44]. To further improve the potential of the harmonization approach, systematic testing of algorithms that also take other parameters into account, could likely further improve functional protein production [7]

In this study it was shown that codon harmonization is a relevant algorithm to include for membrane protein production screens, as it can sometimes lead to significantly higher heterologous production of membrane-embedded proteins compared to other regularly chosen codon variants for heterologous protein production: the wild-type gene or a commercial codon-optimized variant. It seems a relatively robust algorithm as well, as for 5 out of 6 tested membrane proteins, the harmonization algorithm gave either highest production or the production was not significantly different from other high-producing gene variants. However, both the wild-type and optimized variants ended up, for 4 out of 6 tested proteins, among the highest producing variants, which shows there is no single winning variant and several variants can be attempted to improve heterologous membrane protein production.

In conclusion, our results indicate that, the often easily available, wild-type gene for a membrane protein, can often successfully be used in attempts to optimize protein production in *E*. *coli*, when combined with transcriptional tuning, as tuning plays in fact the most important role in improving membrane protein production. However, such an approach could remain unsuccessful; in such case, expressing a codon harmonized variant is a promising method to further attempt to improve membrane protein production. Hereto, we present the Codon Harmonizer as an online-tool to generate such codon harmonized sequences.

## Materials and methods

### Generation of harmonized and optimized gene variants

Codon-optimized sequences were designed using the GeneOptimizer algorithm of GeneArt for expression in *E*. *coli*, avoiding internal restriction sites required for cloning purposes. This algorithm is reported to be a multi-parameter sliding window algorithm, amongst other factors aiming for the usage of frequent codons for the expression host, a good GC-content and the avoidance of repeat sequences [12].

Codon harmonization was performed based on the principle developed before [21,22] and performed with our developed online Codon Harmonizer tool. This tool generates native host and *E*. *coli* codon frequency tables based on complete coding sequence files from full genome assemblies, as deposited at NCBI. These frequency tables are converted to Relative Codon Adaptiveness (RCA) scores, based on the traditional method of Sharp *et al*. [45], however, unlike the original proposal that was based on a limited number of high-expressing genes, here scores are based on all codons of all protein-encoding genes in a genome:

$$RCA=X_{ij}/X_{imax}$$ (1)

In which X_ij_ denotes the number of occurrences of the j^th^ codon for amino acid i and X_imax_ the number of occurrences for the most frequent codon for amino acid i.

These RCA scores were used to find the best matching synonymous codons for the harmonized gene variant (i.e. the synonymous codon with the RCA score in *E*. *coli* closest resembling the RCA score for that codon in the native host). For a limited number of cases, some internal restriction sites had to be removed for cloning purposes by choosing an alternative codon with the second closest RCA.

As a single metric to assess the extent of the harmonization we propose the Codon Harmonization Index (CHI):

$$CHI=\frac{1}{N}\sum_{i=1}^{N}\text{abs}(RCA_{i}-RCA_{i,native})$$ (2)

In which RCA_i_ denotes the relative codon adaptiveness of the i^th^ codon and RCA_i,native_ the relative adaptiveness of the i^th^ native codon of a gene in the native host, and N the number of codons in a gene.

When CHI scores are close to 0, this means the codon landscape of a gene variant is close to that of the native landscape, indicating a well-harmonized codon landscape, the Codon Harmonizer tool in fact tries to minimize the CHI score.

### Codon harmonizer tool

The tool is available for use at http://codonharmonizer.systemsbiology.nl in a Galaxy environment. The stand-alone scripts are written in Python 3.5 and are available at http://gitlab.com/wurssb/codonharmonizer.

### Strains and plasmids

All gene variants were synthesized by GeneArt (Thermo Fisher Scientific). Most synthetic genes were subcloned by GeneArt into the pET28+-based vector pGFPe [39], using XhoI and EcoRI sites. Only for the NorB gene variants XhoI and BamHI were used instead, pGFPe-NorB-wt and pGFPe-NorB-ga were a kind gift from Jan-Willem de Gier [35]. DGGGPs is the only protein in this study with an extracellularly oriented C-terminus, therefore it was cloned into pWarf(+) (Addgene plasmid #34562) instead. This pWarf(+) vector introduces an additional transmembrane domain in between DGGGPs and GFP, allowing for intracellular localization of GFP, required for its maturation and fluorescence [40]. Throughout the study, *E*. *coli* LEMO(DE3) (New England Biolabs) was generally used as an expression strain.

### Culture conditions

Cultivation of *E*. *coli* strains for membrane protein production was generally performed as described before [46]. In short Lysogeny Broth (LB) (5 g/L yeast extract, 10 g/L NaCl and 10 g/L tryptone) was used throughout this study. Antibiotics were added for selection and maintenance of the pET expression vectors (kanamycin (50 μg/mL)) and pLEMO (chloramphenicol (34 μg/mL)).

Fresh transformants of *E*. *coli* were used to inoculate pre-cultures, as the use of re-streaked glycerol stocks may cause severe reduction of expression [46]. Overnight pre-cultures (2 mL in 15 mL Greiner tubes, 37°C, 180 rpm) were used to inoculate 1:50 in 5 mL LB in 50 mL Greiner tubes with different L-rhamnose concentrations (0, 50, 100, 500, 1000 and 2000 μM). At an OD_600_ of 0.35–0.45, cells were induced with IPTG (isopropyl β-D-1-thiogalactopyranoside) at a concentration 0.4 mM. Cells were further incubated for 22 hours after induction (37°C, 180 rpm) and then harvested (13,000x*g*, 10 min, 4°C) for protein production analysis. All data represented are derived from at least 3 independent cultivation experiments.

### Whole-cell GFP fluorescence

Production of membrane proteins was quantified using whole-cell GFP fluorescence as described before [46]. In short, 1 mL of culture was resuspended in ice-cold 100 μL PBS and incubated at 4°C for at least 1 hour for further maturation of GFP. After this, suspensions were centrifuged (10 min, 13,000x*g*, 4°C) and resuspended in 100 μL PBS, which was transferred to a black 96-well microtiter plate with transparent bottoms (PerkinElmer). Fluorescence was directly measured using excitation at 485 nm and emission at 512 nm at a constant gain value (75) (BioTEK SynergyMX).

### In-gel GFP fluorescence

To validate if the GFP signal originates from full-length fusions of the membrane protein with GFP, in-gel fluorescence was performed on the highest expressing samples found by transcriptional tuning, essentially as described before [47,48]. Cell density was determined by measuring the absorbance at 600 nm (OD600) (WPA Biowave). Cultures were centrifuged for 5 minutes at 13,000x*g* and stored at -20°C. After thawing, pellets were resuspended to an estimated final concentration of 0.5 mg protein/100 μL in 50 mM kPi buffer (pH 7.5) (assuming 150 mg protein/L for OD_600_ of 1). This buffer was supplemented with 1 mM MgSO_4_, 10% glycerol, 1 mM EDTA, 0.01 mg/mL DNaseI, 1 mg/mL lysozyme and protease inhibitor (Roche cOmplete^™^, EDTA free). Cells were lysed for one hour under mild shaking at room temperature and stored at -20°C for subsequent analysis. Twenty-five μL 4x Laemmli buffer (Bio-Rad) was added to 75 μL cell lysates, incubated for 5 minutes at 37°C only, as to prevent denaturation of GFP. Directly after resuspension, the samples were shortly sonicated with three consecutive 0.1 ms pulses (Bandelin SONOPLUS HD 3100) to reduce sample viscosity for loading. Twenty-five μL of sample (~90 μg protein) was loaded and separated on a 12% Mini-PROTEAN^®^ TGX^™^ protein gel (Bio-Rad). After electrophoresis, in-gel fluorescence was visualized on a Syngene G-box using a 525nm filter.

## Supporting information

S1 Fig Schematic overview of the *E*. *coli* LEMO21(DE3) system [5,35]. The gene of interest is expressed from a pET vector from a T7 promoter. Transcription is driven by T7 RNA polymerase (T7RNAP), of which the gene is transcribed from an IPTG-inducible promoter, located on chromosomal locus (λDE3 lysogen). The mRNA transcript levels of the gene of interest are tuned by tuning the inhibition of T7RNA; this is accomplished through T7LysY, a T7 lysozyme, inhibiting T7RNAP activity. T7LysY is expressed from the pLEMO and can be tuned by L-rhamnose (*P*_*RhaBAD*_).
(EPS)

S2 Fig Transmembrane helix predictions and codon usage landscapes for the different variants of the membrane proteins in this study. (a) GR; (b) BR; (c) HR; (d) LR; and (e NorB. For the DGGGPs codon landscapes see Fig 1 in the main text. For each protein the upper graphs contain the transmembrane helix prediction plot, which predicts the probability (Y-axis) of residues (X-axis) being in a transmembrane helix domain (red bars), on the inside or cytosolic side of the membrane (blue line) or outside of the membrane (purple line) ((TMHMM v2.0). In the next four graphs for each protein, codon usage landscapes are provided in bars based on Relative Codon Adaptiveness (RCA) scores (Y-axis) for each residue (X-axis) and a moving average (black line) over 5 codons. The first graph (light green bars) gives the codon landscape of the wild-type gene for the native host codon usage, secondly (dark green bars) the codon landscape of the codon-harmonized variant for *E*. *coli* codon usage; thirdly (dark blue bars) the codon landscape of the codon-optimized variant for *E*. *coli* codon usage, fourthly (light blue bars) the codon landscape of the wild-type gene variant for *E*. *coli* codon usage.
(AI) Click here for additional data file.

S3 Fig Membrane protein-GFP fusion integrity check by in-gel fluorescence. Integrity is analyzed for crude cell extracts of *E*. *coli* LEMO21(DE3), expressing the different gene variants at their optimal L-rhamnose concentrations. The Precision Plus Protein^™^ Dual Color marker was loaded on the gels, the fluorescent 25 and 75 kDa bands are indicated (open arrows). The right arrow (filled) indicates the bands most likely containing the protein of interest fused to GFP. It has to be noted that generally membrane protein-GFP fusion bands migrate lower than expected based on their molecular weight, as GFP is still in the folded state. For most variants there is one major fluorescent band representing the membrane protein–GFP fusion, for LR strong fluorescent bands are detected that are probably related to fragmented LR-GFP or non-fused GFP product. Expected sizes full length sizes GR-GFP: 62.3 kDa; BR-GFP: 58.3 kDa; HR-GFP: 55.2 kDa; LR: 64.3 kDa; NorB-GFP: 115.2 kDa; DGGGPs-GFP: 61.0 kDa.
(EPS)

S1 Appendix Gene sequences of wild-type, codon-harmonized and codon-optimized variants of all genes in this work. (TXT)
